# General dentists' knowledge, perceptions, and practices regarding oral potentially malignant disorders and oral cancer in Indonesia

**DOI:** 10.1002/cre2.807

**Published:** 2023-11-27

**Authors:** Elizabeth Fitriana Sari, Wahyu Hidayat, Tenny Setiani Dewi, Sri Rezeki, Rosaline Krimadi, Michael John McCullough, Nicola Cirillo

**Affiliations:** ^1^ Melbourne Dental School The University of Melbourne Carlton Victoria Australia; ^2^ Dentistry Discipline, Department of Rural Clinical Science La Trobe Rural Health School Bendigo Australia; ^3^ Faculty of Dentistry Universitas Padjadjaran Bandung Indonesia; ^4^ Faculty of Dentistry Universitas Syiah Kuala Banda Aceh Indonesia; ^5^ Rumah Sakit Umum Daerah Sele Be Solu I Sorong West Papua Indonesia; ^6^ School of Dentistry University of Jordan Amman Jordan

**Keywords:** GDPs, OC, OPMD, risk factors

## Abstract

**Introduction:**

The most effective means for reducing oral cancer (OC) mortality is by preventing late‐stage disease. Early diagnosis can be improved by increasing awareness among healthcare providers, specifically general dental practitioners (GDP). Therefore, our study aimed to assess GDPs' knowledge of OC risk factors and perceived competence in performing conventional oral examination (COE) in routine dental practice.

**Material and Methods:**

This was a cross‐sectional study conducted in five provinces of Indonesia, namely: Aceh, Banda Aceh (BA); Bandung, West Java (WJ); special district Jakarta (JKT), JKT; Pontianak, West Kalimantan (WK); and Sorong, West Papua (WP). The local Dental Association or Faculty of Dentistry invited the GDPs to attend an education program and complete the survey.

**Results:**

One hundred seventy‐seven GDPs completed the survey (WJ, *n* = 63; BA, *n* = 44, JKT, *n* = 27; WP, *n* = 23; and WP, *n* = 20). A large proportion (164 out of 177, 92.66%) of GDPs felt they had received insufficient training to equip them to diagnose OC and as many as 22.6% (*n* = 40) did not refer to specialists when they found suspicious mucosal lesions. Notwithstanding the significant regional variations, the majority of Indonesian GDPs self‐reported inadequate knowledge and awareness of OC and scarce confidence in performing COE.

**Conclusion:**

GDP knowledge of OC risk factors and COE is key to improving early diagnosis of OC at a community level. Therefore, it is suggested that the lack of knowledge and confidence of GDPs reported here should be addressed through the national dental curriculum in Indonesia.

## 1 INTRODUCTION

Detection of subjects with oral potentially malignant disorders (OPMDs) may facilitate an early diagnosis of oral cancer (OC), with consequent reduction of cancer burden (Mortazavi et al., 2014). Late‐stage OC is associated with significant morbidity and mortality. In contrast, early‐stage OC has a favorable prognosis, with a 5‐year survival rate of 66%–85% of individuals without regional lymph node involvement (Sciubba, 2001). Disappointingly, almost half of the patients with OC experience diagnostic delay and over 50% present with advanced‐stage disease (Gonzalez‐Moles et al., 2022). Thus, early diagnosis of OC is key, with the detection of an OPMD an important preventive measure to reduce the morbidity and mortality for OC (Warnakulasuriya et al., 2007). The estimated global prevalence of potentially malignant disorders of the oral cavity is approximately 1%–5% (Petti,^2003^), and these share some common OC risk factors such as smoking and betel quid chewing.

Unfortunately, no accurate data exists on the prevalence of OPMD in Indonesia. Rates of OC are also uncertain given the lack of a comprehensive national cancer registration system. Therefore, we previously conducted a pilot study to perform OC screening in Indonesia, where OPMDs were detected in 11.12% of participants and 0.2% had oral malignancy (Sari et al., 2017). More recently, the Global Burden of Disease (GBD) study reported an age‐standardized incidence rate of lip and oral cavity cancer of 5.3 per 100,000 for Indonesia, which is likely an underestimation of the magnitude of the problem (Sari et al., 2023). One‐third of Indonesians are tobacco smokers (about 87.3 million people) (Nurhasana et al., 2020; Soendoro, 2013; Wibowo et al., 2019), and areca nut chewing habits are a “heritage culture” passed down through many generations (Cheong et al., 2017; Fotlona, 2013). These lifestyle practices result in Indonesians having a high risk of developing malignancy, driving an increase in the incidence of OPMD and OC.

Several studies have reported that developed countries have relatively low rates of OC, while developing countries show higher rates in both females and males (Ferlay et al., 2010; Sharma & Om, 2015). Further, improved health system infrastructure has the potential to curb OC mortality worldwide (Jemal et al., 2010; Sankaranarayanan et al., 1998). The increasing number of OC cases and unimproved 5‐year survival rate, therefore, could be due to several factors such as people's and practitioner's scarce awareness of OC.

General dental practitioners (GDPs) play a vital role in the detection of oral malignant and OPMDs. However, many GDPs fail to assess or diagnose OC and are not trained to detect early precursor lesions. The obstacles in undertaking a routine oral examination by GDPs have been recognized and include practitioners' lack of knowledge and experience (Gómez et al., 2010; Varela‐Centelles et al., 2015). In addition, inadequate GDPs' skills, knowledge, and confidence to detect mouth cancer and precursor lesions may result in insufficient preventive education provided to their patients (Ford & Farah, 2013). GDPs' knowledge and awareness in recognizing early precursor lesions and educating patients, their ability to perform a complete examination and to appropriately refer patients have been shown to improve diagnostic delay and to decrease case fatality (Crossman et al., 2016; Gigliotti et al., 2019; Sankaranarayanan et al., 2002; Yellowitz & Goodman, 1995).

Given the importance of GDPs in OC early diagnosis and the lack of data regarding current practices of Indonesian GDPs, a study assessing their knowledge and awareness of OC and OPMD was warranted. We hypothesized that if a sizeable number of GDPs self‐reported inadequate skills and confidence in performing OC early screening, a training intervention on OC screening could be beneficial to enhance GDP's knowledge, confidence, and ability to detect OPMD and OC earlier.

In the present study, we conducted a survey in diverse regions of Indonesia, namely Aceh, West Papua (WP), West Java (WJ); West Kalimantan (WK); and Central District of Jakarta (JKT). The aim of our study was to assess GDPs' knowledge of OC risk factors and their perceived competence in performing OC early screening in routine dental practice. The questions in the survey included GDPs' self‐reported confidence in performing OC screening examination, knowledge of recognizing risk factors, and early signs of OC or OPMD.

## 2 METHODS

### 2.1 Study design

This was a descriptive cross‐sectional study using a questionnaire survey. We adopted a multistage, stratified, cluster‐sampling procedure, which considered geographical region, degree of urbanization, economic development status, gender and age distributions. The five cities selected for the study were: Banda Aceh (BA, Aceh), Bandung (WJ), JKT (special city district of Indonesia), Pontianak (WK), and Sorong (WP).

### 2.2 Participant recruitment and sampling

The local Dental Association or Faculty of Dentistry invited the GDPs to attend a 1‐day education program on oral malignant and OPMDs detection. The GDPs who attended the meeting obtained a clear explanation about the study and were given a copy of the plain language statement. The GDPs who consented were enrolled in the study after completing and signing an informed consent form. Consecutive sampling was used as a technique in recruiting respondents and involved selecting all individuals who agreed to participate, provided they met pre‐established criteria, until the desired number of subjects was recruited.

### 2.3 Questionnaire

The questionnaire consisted of 43 questions developed from existing literature and modified to suit the local situation (Farah & McCullough, 2008; Mao, 2012; Maybury et al., 2012; Morse et al., 2011; Varela‐Centelles et al., 2015). It underwent a validation process by a panel of senior researchers based in Indonesia to critically evaluate the suitability, clarity, simplicity, understanding, and sequencing of the questions (Appendix A). Following a pilot study that consisted of 10 Indonesian GDPs, appropriate modifications to the questionnaire were made before it was utilized in the main survey. The questionnaire consisted of multiple choice, open and closed questions, Likert scale including GDP's knowledge, awareness, attitude, subjective assessment of their clinical competence for screening of OC, and level of education.

### 2.4 Approvals

Ethical approval was obtained from the Faculty of Medicine University of Padjadjaran, Health Research Ethic Committee, Indonesia (reference number 701/UN6.C1.3/KEPK/PN/2016), and from the University of Melbourne (Ethics reference ID: 1748812). Furthermore, formal arrangements were made with Indonesian local dental associations (*Persatuan dokter gigi Indonesia*) and the Faculty of Dentistry.

### 2.5 Data analysis

Completed questionnaire data were stored in the SurveyMonkey application. Data were exported to Microsoft Excel and were deidentified and coded to allow statistical analysis using Minitab Express© version 1.5.1. 2017 and R commander. The difference between means was determined using multiway analysis of variance (ANOVA) followed by *χ* ^2^ and analyzing Likert scale results with logistic regression. Differences were considered significant where *p* < .05.

## 3 RESULTS

### 3.1 Demographics of GDPs who participated in the study

The number of GDPs who enrolled in the education program and filled in the questionnaire was 177 (Table 1). The highest number of registered GDPs was from WJ region (*n* = 63), followed by BA (*n* = 44), JKT (*n* = 27), WP (*n* = 23), and WP (*n* = 20). Female respondents were predominant (*n* = 157, 88.7%).

**Table 1:** Distribution of GDPs' workplace in each region studied.

Workplace	WK		WP		WJ		JKT		BA		All regions	
	*N*	%	*N*	%	*N*	%	*N*	%	*N*	%	*N*	%
Community service	11	47.8	8	40	59	93.7	3	11.1	8	18.2	89	50.3
Dental practice	3	13	8	40	2	3.2	17	63	18	40.9	48	27.1
Dental hospital	4	17.4	3	15	0	0	6	22.2	4	9.1	17	9.6
More than one places	5	21.7	1	5	2	3.2	1	3.7	14	31.8	23	13
ALL	23	100	20	100	63	100	27	100	44	100	177	100

Abbreviations: BA, Banda Aceh; GDP, general dental practitioner; JKT, Jakarta; WJ, West Java; WK, West Kalimantan; WP, West Papua.

More than half (50.28%) of the GDPs who enrolled in this study worked in community service. Slightly over a quarter (*n* = 48, 27%) of respondents worked in private dental practice, 9.6% (*n* = 17) worked in the dental teaching hospital, and the remainder worked in more than one location.

GDPs' workplace varied considerably depending on the region examined. In the WJ region, 93.85% GDPs who enrolled in this study worked in community service, whereas only 47.83% and 40% GDPs from WK and WP worked in community service, respectively. Conversely, most GDPs from JKT region (62.96%) and BA (40.91%) worked in private dental practice (Table 1).

### 3.2 GDPs' awareness of OC in different Indonesian regions

GDPs' response toward questions related to their awareness of OC and OC training received at dental schools from each region varied considerably (Table 2). A large proportion (164 out of 177, 92.66%) of GDPs claimed to have received insufficient training to equip them to diagnose OC. All GDPs from WP (*n* = 20, 100%) felt they had insufficient training. A sizeable number of GDPs (8 out of 44, 18.18%) from BA felt they had received sufficient OC training at dental school (Table 2).

**Table 2:** GDPs' response regarding OC in selected study regions.

Question	Response	WK		WP		WJ		JKT		BA		Total		*p* Value
		*N*	%	*N*	%	*N*	%	*N*	%	*N*	%	*N*	%	
Training OC at Dental School	Insufficient	22	95.7	20	100	61	96.8	25	92.6	36	81.8	164	92.7	<.05
	Sufficient	1	4.4	0	0	2	3.2	2	7.4	8	18.2	13	7.3	
Educated patient of OC Risk factor	No	14	60.9	18	90	57	90.5	23	85.2	32	72.7	144	81.4	<.05
	Yes	9	39.1	2	10	6	9.52	4	14.8	12	27.3	33	18.6	
Perform Screening in high‐risk patients	No	11	47.8	15	75	39	61.9	11	40.7	15	34.1	91	51.41	<.05
	Yes	12	52.2	5	25	24	38.1	16	59.3	29	65.9	86	48.7	
Perform complete examination	No	8	34.78	15	75	40	63.5	15	55.6	31	70.5	109	61.7	<.05
	Yes	15	65.2	5	25	23	36.5	12	44.4	13	29.6	68	38.4	
Refer if OPMD or OC observed?	No	3	13.04	6	30	7	11.1	10	37.0	14	31.8	40	22.6	<.05
	Yes	20	86.9	14	70	56	88.8	17	62.9	30	68.2	137	77.4	
Total		23	100	20	100	63	100	27	100	44	100	177	100	

*Note*: 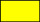, significantly different for entire row (Insufficient vs Sufficient, No vs Yes).

Abbreviations: BA, Banda Aceh; GDP, general dental practitioner; JKT, Jakarta; OC, oral cancer; OPMD, oral potentially malignant disorder; WJ, West Java; WK, West Kalimantan; WP, West Papua.

An overwhelming majority of GDPs (81.36%) admitted that they failed to educate their patients on OC risk factors. Eighteen out of 20 (90%) GDPs from WP reported to neglect educating patients and 90.48% GDPs from WJ did not educate patients either.

Over the whole five regions, 51.41% of GDPs indicated that they did not perform a screening on high‐risk patients. In each region, there was some variation with 75% (15/20) and 61.9% (39/63) GDPs from WP and WJ reportedly skipping OC screening to high‐risk patients. Conversely, 65.91%, 52.17%, and 59.26% GDPs from BA, WK, and JKT regions, respectively, undertook OC screening in high‐risk patients. Similar responses were obtained for complete mouth examination (Table 2). Interestingly, 70.45% GDPs from BA region did not routinely perform a complete mouth examination, even though 65.91% reportedly undertook screening of high‐risk patients. This potentially contradictory response makes the screening results in high‐risk patients questionable.

### 3.3 Referral patterns

Overall, 22.6% (*n* = 40) of GDPs choose not to refer to specialists when they find suspicious mucosal lesions. 13% (3 out of 23) and 30% GDPs (6 out of 20) from WK and WP, respectively, reported not to refer the patient with mucosal lesions. Furthermore, there were also few GDPs from urbanized regions, such as WJ (16 out of 63) and JKT (16 out of 27), who also elected not to refer patients with oral mucosal lesions despite the presence. Upon questioning regarding referral patterns after the recognition of suspected oral mucosal lesions, more than half of the GDPs (54.8%) indicated that they would refer to oral medicine (OM) specialists if they suspected their patients had either an OC or OPMD (Table 3). A sizeable number (*n* = 54, 29.94%) of GDPs did not know who best to refer their patients. 13.56% decided to send the patient to oral surgery and 1.13% to oral pathologist. As high as 75% (15 out of 20) of GDPs from WP responded that they do not know who to refer their patients. 59.26% (16 out of 27) GDPs from JKT also responded they were confused as to whom to send the patients for further treatment. Conversely, all GDPs from BA reported knowing where to refer the patients, and most (90.91%) would send patients suspected with OC or OPMD to an OM specialist.

**Table 3:** Referral patterns.

Referral patterns	WK		WP		WJ		JKT		BA		All regions		*p* Value
	*N*	%	*N*	%	*N*	%	*N*	%	*N*	%	*N*	%	
I don't know	6	26.1	15	75	16	25.4	16	59.3	0	0	53	29.9	<.05
Oral medicine	15	65.2	1	5	32	50.8	9	33.3	40	90.9	97	54.8	
Oral surgery	2	8.7	4	20	14	22.2	1	3.7	3	6.8	24	13.6	
Oral pathologist	0	0	0	0	1	1.6	1	3.7	0	0	2	1.1	
Oncologist	0	0	0	0	0	0	0	0	1	2.3	1	0.6	
All	23	100	20	100	63	100	27	100	44	100	177	100	

*Note*: 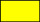, significantly different.

Abbreviations: BA, Banda Aceh; GDP, general dental practitioner; JKT, Jakarta; WJ, West Java; WK, West Kalimantan; WP, West Papua.

### 3.4 GDPs' ability to perform COE and operative diagnostic techniques

There are eight steps to follow for a complete oral examination. The majority of GDPs claimed they performed COE steps, with the majority performing a gingival examination (90.98%), followed by a buccal (82.49%), labial (76.27%), lips (76.27%), and tongue (54.24%) examination. Very few GDPs performed bimanual and floor of the mouth examinations (22.03%) (Table 4). Only 2.84% (*n* = 5) GDPs from five selected provinces reported the ability to perform oral mucosal biopsy, 1.69% (*n* = 3) claimed to have the ability in applying toluidine blue staining procedure, and 2.26% (*n* = 4) the use of an adjunct light‐based tool in OC early detection (Table 5).

**Table 4:** COE steps done by GDPs.

COE steps	No	Yes
	*N* (%)	*N* (%)
1. Perform extra‐oral examination	38	139
	21.47	78.53
2. Perform lips examination	42	135
	23.73	76.27
3. Perform labial examination	51	126
	23.73	76.27
4. Perform buccal examination	31	146
	17.51	82.49
5. Perform gingival examination	16	161
	9.04	90.96
6. Perform palatal examination	43	134
	24.29	75.71
7. Perform tongue examination	81	96
	45.76	54.24
8. Perform bimanual and FOM examination	138	39
	77.97	22.03

Abbreviations: COE, conventional oral examination; GDP, general dental practitioner.

**Table 5:** GDPs' ability to perform biopsy and adjunct tool.

No	Additional information	Yes (%)	No (%)
1	Have ability to perform oral mucosal biopsy	5	171
		2.84	97.16
2	Have ability to perform toluidine blue staining	3	174
		1.69	98.31
3	Have ability to use light‐based adjunct tools (e.g., Velscope®)	4	173
		2.26	97.74

## 4 DISCUSSION

In the present study, we surveyed GDPs from five Indonesian provinces to assess their knowledge, perceptions, and practices regarding OPMDs and OC. Overall, our results show that GDPs feel they are not adequately equipped to prevent, diagnose, and manage OC patients. Most GDPs fail to educate their patients about modifiable risk factors and do not perform screening in high‐risk patients. With regional variations, a sizeable number of GDPs do not refer patients with suspected OPMD and OC.

This was the first study surveying a sample of the Indonesian dentists across several provinces. More than half (50.28%) of the GDPs in the present study worked in community service or primary health care. The community service is the front‐line health facility funded by the Indonesian government, where most individuals from middle and lower socioeconomic groups primarily seek care due to government subsidizing payment. Indonesian community services often lack good infrastructure, financial and human resources (Lazuardia et al., 2013; Rahardjo & Maharani, 2014). Dental practice and hospital are the second choice for people as they are much more expensive compared to community service. In remote areas, such as WP and WK, community service has a vital role for oral health. Most GDP respondents from JKT region (62.96%) and many from BA (40.91%) worked sometime in private dental practice, with only a few GDPs working exclusively in dental hospitals or general hospitals.

In relation to OC curriculum training that was offered during their dental studies, the majority of GDP respondents in the present study felt they had received insufficient training on OC. This should be taken into consideration by the relevant planning and regulatory bodies, and the Indonesian Faculty of Dentistry may consider implementing OC training to allow GDPs to have adequate knowledge, skill, attitude, and confidence in a routine OC examination. Dental practitioners should remain aware of early sign and symptoms of cancer (Farah & McCullough, 2008; Varela‐Centelles et al., 2015) as visual and tactile examination of a conventional oral examination that could detect early oral carcinoma (Irwin et al., 2011; Rethman et al., 2010; Varela‐Centelles et al., 2015).

As part of OC prevention, educating patients regarding OC risk factors, particularly the major risk factors such as tobacco, areca nut, and alcohol, is of paramount importance. Also, it is desirable that patients receive adequate information about OC risk factors, especially from dental practitioners (Farah & McCullough, 2008; Maybury et al., 2012; Varela‐Centelles et al., 2015). Disappointedly, our study revealed that most GDPs failed to educate patients on preventable lifestyle habits and behaviors that constitute OC risk factors. This has the potential to have a tremendous impact on OC incidence and prognosis in Indonesia.

More than half GDPs from the five studied regions admitted not performing a screening of high‐risk patients. Many GDPs who worked in the front‐line community service did undertake screening; however, they reported not to be sufficiently confident in performing these procedures. Notably, GDP/population ratio in Indonesia is 3.4 per 100,000 people (Rahardjo & Maharani, 2014; Soendoro, 2008, 2013). The limited number of GDPs in Indonesia poses considerable challenges to the oral healthcare system (Lazuardia et al., 2013; Soendoro, 2013). This shortage of human resources and the high demand for primary care might be a main reason for GDPs not performing a routine complete examination, particularly in high‐risk patients.

Almost a quarter of GDPs do not refer to specialists when they observe suspicious lesions, potentially due to confusion as to whom they should refer. Remote regions in Indonesia have limited health facilities and specialist clinicians. However, there were also GDPs from WJ, BA, and JKT who did not routinely refer patients with OPMD or OC. This is more controversial as these regions are not considered remote and have more extensive health facilities compared to remote areas such as WK and WP regions. This could be due to a related lack of knowledge as to where best to refer for further treatment, but it is also possible that GDPs are not confident in diagnosing oral mucosal diseases and, when they suspect OPMD/OC, are not familiar with the appropriate referral pathways for these patients (BDA, 2000; Mao, 2012; Morse et al., 2011; Varela‐Centelles et al., 2015).

OM specialists were GDPs' number one choice for referral of patients with suspected OC or OPMD. However, the number of OM specialists in Indonesia is limited, with only approximately 183 specialists who serve over 275 million Indonesians (ISPMI, 2023). The scarcity of OM practitioners may be due to the exiguous training opportunities, with only three institutions across Indonesia offering OM training programs, the University of Indonesia in JKT, the University of Padjadjaran in WJ, and the University of Airlangga in Surabaya. All of these institutions are concentrated in Java island (ISPMI, 2023). Currently, there are two OM specialists reported to work in WP, but no oral pathologists (ISPMI, 2023). Our own fieldwork experience in WP suggests that certain OC‐related health facilities, such as pathology clinics, are lacking in WP, and all biopsy specimens need to be sent to other islands, such as Sulawesi or Java, for processing.

GDPs from JKT also reported some confusion as to whom they should refer their patients for further treatment. In general, at a national level, there are only 3 dental specialists per 10,000 Indonesians (Rahardjo & Maharani, 2014; Soendoro, 2008), hence there is a need to improve dental health system and in particular the number of human resources.

The shortage of specialists together with dentists' privileged position as frontline dental professionals means that the burden of OC detection rests mostly with GDPs. The good news is that OC screening can be very effective with a simple visual examination (Sciubba, 2001). This can occur at a routine dental check or, in underserved areas, by screening that targets high‐risk populations (Dwivedi et al., 2023). Sadly, research shows that general dentists feel ill‐equipped and perform poorly when it comes to addressing suspicious lesions (Grafton‐Clarke et al., 2019), and our study is in line with previous research. It would seem sensible, therefore, that the fight to reduce oral cancer mortality starts with a reform of dental and oral health curricula. In fact, lack of GDP's knowledge, skill, and awareness of performing OC screening is a common problem worldwide. Therefore, we suggest improving oral medicine curriculum with a more operative‐clinical approach, where students can ideally assess more real‐life cases directly and perform COE first hand. Further, case‐based approach in the form of case‐initiated and problem‐based learning could increase students' understanding and skills in analyzing oral diseases.

In particular, COE should be included in a routine comprehensive examination that targets not only OC, but also oral diseases as a whole. The eight steps of COE by visual and tactile examination should be part of a routine clinical assessment in GDPs' daily practice. The present study shows that whilst gingival examination was routinely undertaken, bimanual and floor of the mouth examinations were not. About half GDPs neglected tongue examinations. This is a worrisome finding, as these two areas are at high risk for the development of OPMD and could potentially develop into carcinoma (Mortazavi et al., 2014; Sreedhar et al., 2015; Warnakulasuriya et al., 2007). Hence, improvements are needed in the dental curriculum to equip students to undertake a proper clinical assessment of the oral mucosa and to recognize mucosal abnormalities, including OPMD and cancer. It is important to acknowledge, however, that there could be local and regional differences and/or specific areas of weakness in the dental curriculum and professional development. For example, a recent study assessing knowledge and practice regarding OC among dentists in JKT (Wimardhani et al., 2021) found that GDPs had a considerable level of knowledge of major risk factors of OC. However, the study highlighted that there were gaps especially in their perceived ability to perform diagnostic procedures. This calls for action, particularly in improving dentists' competencies in operative procedures as well as background knowledge.

## 5 CONCLUSION

GDPs have an important role in OC screening, however, most Indonesian GDPs surveyed appear not to have adequate knowledge of OC risk factors as well as inadequate knowledge, awareness, and confidence in performing COE as part of their routine clinical examination. This study also identified the most likely obstacles related to what specific steps the GDPs tended to avoid and why they were not confident in managing patients with OPMD/OC. Based on the response received, it would be sensible to promote a programmatic discussion with the relevant stakeholders and policy makers, including the Indonesian government and the Faculty of Dentistry, with the aim of incorporating OC training in the national dental curriculum.

## AUTHOR CONTRIBUTIONS

*Study conception and design*: Elizabeth Fitriana Sari and Nicola Cirillo. *Data collection*: Elizabeth Fitriana Sari, Wahyu Hidayat, Tenny Setiani Dewi, Sri Rezeki, and Rosaline Krimadi. *Subsequent analysis*: Elizabeth Fitriana Sari, Michael John McCullough, Nicola Cirillo. *Interpretation of results*: Elizabeth Fitriana Sari, Michael John McCullough, Nicola Cirillo. *Supervising the project*: Michael John McCullough and Nicola Cirillo. *Funding acquisition*: Elizabeth Fitriana Sari and Nicola Cirillo. All authors contributed in drafting the manuscript or revising it critically for important intellectual content and approval of the final version to be published and agreement to be accountable for the integrity and accuracy of all aspects of the work.

## CONFLICT OF INTEREST STATEMENT

The authors declare no conflict of interest.

## Data Availability

The data that support the findings of this study are available from the corresponding author upon reasonable request

## APPENDIX A QUESTIONNAIRE FOR GDPS

### A.1

**Personal Data^a^**

Name:

Qualification: GDP/Specialist________ (Please circle, and fill in the blank if others)

Date of Birth:

Institution: Private clinic/Hospital/Primary health care (Please circle)

Address:

Email:

Phone:

^a^ Personal data will be treated confidentially.

Please kindly answer the questions below. You may answer in Bahasa Indonesia or English.

1. Have you ever undertaken an oral cancer screening examination?

a. Yes
b. No

2. Do you always perform a complete oral mucosal examination to every patient?

a. Yes
b. No

3. Have you ever found patients with suspected oral malignant lesions?

a. Yes, *often* (please roughly state the number of times each year__________________)
b. Yes, *seldom* (please roughly state the number of times each year__________________)
c. Never

4. Have you ever found patients with suspected oral premalignant lesions?

a. Yes, often (please roughly state the number of it in a year__________________)
b. Yes, seldom (please roughly state the number of it in a year_________________)
c. Never

5. Do you regularly advise your patients about oral cancer risk factors?

a. Yes
b. No

6. Do you regularly encourage patients to reduce oral cancer risk factors?

a. Yes
b. No

7. Do you believe that you have sufficient knowledge concerning prevention of oral cancer?

a. Yes
b. No

8. If not routine, do you perform oral cancer screening in high‐risk patients?

a. Yes
b. No

9. Do you believe you have sufficient training, knowledge, and technique to perform oral cancer detection?

a. Yes
b. No

10. Do you believe you have sufficient training, knowledge, and technique to detect oral premalignant lesions or early oral cancer lesions?

a. Yes
b. No

11. Do you believe that you have received sufficient training on oral cancer at Dental school?

a. Yes
b. No

12. Please indicate below what you believe as risk factors of oral cancer.
13. Please indicate below any pathologic changes in oral cavity associated with oral cancer.
14. Please indicate below any pathologic changes in oral cavity associated with oral malignant lesions.
15. Do you have the required equipment to perform an oral cancer screening?

a. Bright white light (Yes/No)
b. Dental Mirror (Yes/No)
c. Gauze square (Yes/No)

16. Do you perform extra‐oral examinations?

a. Perform
b. Do not perform

17. How confident are you in undertaking an extra‐oral examination?

a. Very confident
b. Confident
c. Not confident
d. Very not confident

18. How confident are you in identifying pathologic changes on extra‐oral?

a. Very confident
b. Confident
c. Not confident
d. Very not confident

19. Do you perform lip examinations?

a. Perform
b. Do not perform

20. How confident are you in undertaking a lip examination?

a. Very confident
b. Confident
c. Not confident
d. Very not confident

21. How confident are you in identifying pathologic change on lips?

a. Very confident
b. Confident
c. Not confident
d. Very not confident

22. Do you perform labial examinations?

a. Perform
b. Do not perform

23. How confident are you in undertaking a labial examination?

a. Very confident
b. Confident
c. Not confident
d. Very not confident

24. How confident are you identifying pathologic changes on labial mucosa?

a. Very confident
b. Confident
c. Not confident
d. Very not confident

25. Do you perform buccal mucosa examinations?

a. Perform
b. Do not perform

26. How confident are you in undertaking a buccal mucosa examination?

e. Very confident

a. Confident
b. Not confident
c. Very not confident

27. How confident are you in identifying pathologic changes on buccal mucosa?

a. Very Confident
b. Confident
c. Not confident
d. Very not confident

28. Do you perform gingival examinations?

a. Perform
b. Do not perform

29. How confident are you in undertaking a gingival examination?

a. Very confident
b. Confident
c. Not confident
d. Very not confident

30. How confident are you in identifying pathologic change on gingival?

a. Very Confident
b. Confident
c. Not confident
d. Very not confident

31. Do you perform a ventral tongue and floor of the mouth examinations?

a. Perform
b. Do not perform

32. How confident are you in undertaking a ventral tongue and floor of the mouth examination?

a. Very confident
b. Confident
c. Not confident
d. Very not confident

33. How confident are you in identifying pathologic changes on ventral tongue and floor of the mouth?

a. Very Confident
b. Confident
c. Not confident
d. Very not confident

34. Do you perform a bimanual palpation on floor of the mouth?

a. Perform
b. Do not perform

35. How confident are you in undertaking a bimanual palpation floor of the mouth examination?

a. Very confident
b. Confident
c. Not confident
d. Very not confident

36. How confident are in identifying pathologic changes on floor of the mouth?

e. Very Confident

a. Confident
b. Not confident
c. Very not confident

37. Do you believe you have the ability to perform an oral mucosal biopsy?

a. Yes,
  If yes, have you performed an oral mucosal biopsy? Please circle the answer (Yes/No)
b. No

38. Do you believe that you have the ability to perform Toluidine blue staining?

a. Yes, where did you learn? please choose:

- Dental school
- Course
- Others, please specify_____________________

b. No

39. Do you believe that you have the ability to use the adjunct tool for oral pre‐cancer/cancer detection such as Velscope?

a. Yes, where did you learn? Please choose:

- Dental school
- Course
- Others, please specify_____________________

b. No

40. Have you done oral disease detection utilizing Velscope or any other tools? Please circle

a. Yes, please specify____________________
b. No

41. Will you refer your patients suspected to have oral malignant lesion?

a. Yes
  If yes, where will you refer them to? Please circle the answer (to Oral Medicine Specialist/oral Surgery/oral Pathology/else _________________)
b. No, as I have never suspected any of my patients to have had an oral malignant lesion.

42. Will you refer your suspected patients with oral pre‐malignant lesions?

a. Yes
  If yes, where will you refer them to? Please circle the answer (to Oral Medicine Specialist/oral Surgery/oral Pathology/else _________________)
b. No, as I have never suspected any of my patients to have had oral pre‐malignancy.

43. What will you prescribe at the first appointment when you suspect a patient has an oral malignant or pre‐malignant lesion?

a. Mouthwash
b. Analgesic
c. Antibiotic
d. Antiviral
e. Do not give any medication and only do referral
f. Other, please specify____________________________
